# Do executive function and family factors predict children’s preference for trendy over classic toys? An experimental investigation

**DOI:** 10.3389/fpsyg.2023.1190876

**Published:** 2023-06-16

**Authors:** Margarita Gavrilova, Vera Sukhikh, Nikolay Veresov

**Affiliations:** ^1^Faculty of Psychology, Lomonosov Moscow State University, Moscow, Russia; ^2^School of Education Culture & Society, Monash University, Melbourne, VIC, Australia

**Keywords:** child psychology, cultural-historical approach, play, trendy toys, toy industry, toy preference, executive functions

## Abstract

In the last decade, global trends and social media have covered almost the entire world, including children population. The toy industry is filled with new items whose popularity has been triggered by viral publications on social networks or by popular animated films. The present experimental study is the first to (i) describe the characteristics of toy choice in the context of the spread of global trends, and (ii) examine the influence of family and individual child characteristics on the dependence on trends in toy choice. The sample consisted of *N* = 127 children aged 3–4  years. Children had individual assessment of non-verbal intelligence main executive function skills (cognitive flexibility, working memory and inhibition control) and took part in the experiment, while their parents completed a questionnaire on family background. Analysis of children’s answers about the reason for choosing a particular toy indicates uncertain motivation in choosing a trendy toy in contrast to the classic ones. It is reflected in the fact that children do not know what exactly and how they will play with it. It is revealed that boys are 1.66 times more likely to prefer the trendy toy than girls. It was also found that as inhibitory control increased, children were less dependent on tendencies to choose toys.

## 1. Introduction

A toy is both an object of children’s play, an object of culture, and an object of the global toy industry (Francis, 2010; Mertala et al., 2016). Although children’s play and culture have relatively stable characteristics over time, the children’s consumer culture been changing rapidly over the past decade. The digitalization of the toy industry leads to an instant spread of trends that are displacing more traditional children’s toys (Marsh, 2017). For example, children’s plays are increasingly based on the plots of popular animated films and video games (Klinger, 2007; Hinkley et al., 2018). Such animated products are entering children’s playing as a result of the active marketing and commercialization of the toy industry.

From the perspective of the cultural-historical approach to the understanding of children’s play, there are strong theoretical reasons to suggest that the spread and uncontrolled change of trendy toys can lead to negative consequences for children’s development (Smirnova, 2015; Ryabkova et al., 2019; Veraksa et al., 2020). First, the toy is the material for framing children’s play and creating imaginative situations (Elkonin, 1980, 2005; Vygotsky, 2004; Francis, 2010; Wynberg et al., 2022). As a result of increased interest in animated film and video game plots, children actually lose the opportunity to construct a story on their own and often find it difficult to move away from the plots set by the film or game. Second, a story-rich and meaningful play promotes children’s development. Play effectively trains executive function skills (Kelly et al., 2011; Fleer et al., 2019; Veraksa et al., 2020b; Vidal Carulla et al., 2021; Doebel and Lillard, 2023), emotional and social competencies (Mathieson and Banerjee, 2011; Howard et al., 2017; Colliver and Veraksa, 2021), and language skills (Nicolopoulou et al., 2015; Quinn et al., 2018). The prevalence of animated film and play plots strips children’s play of essential features such as spontaneity, initiative, and freedom. Thirdly, the toy helps the child to reproduce certain situations and cultural contexts in play. That is, play is a symbolic means of understanding the surrounding world for the child (Smirnova, 2015). In recent decades children have been losing interest in reproducing real-life events and cultural themes in play (tea parties, mother–daughters, playing in a hospital). Thus, children are to some extent losing the opportunity to learn about real-life natural and social processes through play, as their attention is occupied by trendy toys.

Trendy toys are toys that become extra popular for a period of time. Over the past few years, the biggest trends in toyse were Spinner and Pop It, whose popularity has been driven by viral videos on social media. The 2022–2023 Huggy Wuggy is a new trendy toy that has gained popularity worldwide. Huggy Wuggy is not a classic harmless toy: it is a sinister monster from a survival horror game called Poppy Playtime. According to the plot of this game, a plush toy with sharp teeth singing about “hugging and killing” stalks the player to kill him. In the short period of the Huggy Wuggy toy’s explosive popularity, there is a wealth of evidence to suggest that it has a negative impact on children’s psychosocial development. For example, a study by Pelli (2022) on children’s fears describes a case of severe fear of the Huggy Wuggy. The child drew a picture of crawling under the bed so that the monster would not see and eat him.

## 2. Current study

The present study is an experiment based on the ideas of the cultural-historical approach. The aim of this study was to determine the predictors that are related to children’s trend dependency in choosing toys. The study covered three research questions: (i) How do children choose toys and how do they explain their choices in relation to the classic and trendy toys? (ii) whether there is any impact of family factors (SES, parents’ education and number of children) on children’s trend dependency in their choice of toys; (iii) do EF skills predict a children’s trend dependency in choosing toys?

The theoretical background for this research is related to key theoretical and methodological ideas of the cultural-historical approach. Based on the work of Vygotsky it follows that a toy should help a child to experience an imaginary situation and to accept a play role and its associated rules (Vygotsky, 2004). Hence, as a result of the experiment it is expected that children will choose preferably those toys which allow to create a rich imaginary situation. This requires children to have an idea of possible play actions and plots which can be implemented in play with a particular toy. In addition, Elkonin showed that the object of children’s play is most often the relationship between people (Elkonin, 1980). Thus, as a result of the experiment children are expected to choose toys that represent the meaning and content of people’s relationships.

Furthermore, it is expected that children with weak EF skills will show higher trend dependency in choosing toys. The theoretical justification behind this assumption is that EF skills on the one hand provides an opportunity for play (as well as any other complex self-directed activity; Kelly et al., 2011; Veraksa et al., 2019), but also develops through meaningful true play (Veraksa et al., 2020; Vidal Carulla et al., 2021). The choice of a trendy toy will indicate that the child is not looking for the possibility of creating imaginative situations and playful plots in the toy, but is primarily guided by its appearance and popularity.

## 3. Materials and methods

### 3.1. Procedure

Data were collected between October and December 2022. During this time in the country of study the virally popular trendy toy was Huggy Wuggy. This toy is described with more detail in the Introduction section. Due to its off-trend popularity among pre-school and school-age children, Huggy Wuggy (hereinafter Trendy Toy) was the one used in this study to assess children’s trend dependency in choosing toys.

The research procedure included two stages. In the first stage, an individual assessment of the EF skills was carried out by experienced testers. The tools were administered in a strictly order. Each meeting with a child lasted no more than 15 min.

In the second stage, an individual experimental session within the framework of a forced choice paradigm was conducted to explore children’s toy preferences. It consisted of two steps. In the first step, the child was shown a specially selected set of 12 toys. These were the toys that could interest children with different interests and preferences: сar, dog, teddy bear, girl doll, male doctor doll, hospital playset, supermarket playset, space robot, fairy dragon, human family, family of tigers, family of cartoon animals. Toys were sampled based on the expression of characteristics according to four criteria: genderness (‘girly’ toy, ‘neutral’ toy and ‘boy’ toy); detailedness (three toys from minimally detailed to maximally detailed); realism (three toys from minimally realistic to maximally realistic); anthropomorphism (three toys from minimally anthropomorphic to maximally anthropomorphic). They were placed on a white cloth in a certain order and at a given distance from each other. Each toy was shown to the child and named. Then a child was asked to choose one of them that he/she would like to play with the most and tell why. Next, the child was shown two toys: the choice toy and the Trendy Toy. The child was again asked to choose one of these two toys, which one he wanted to play with the most. If the child chose the Trendy Toy trendy toy, he/she was asked to tell why he/she chose it.

### 3.2. Participants

A total of 127 children (51% girls) participated in the study. The children were between 3 and 4 years old at the time of the study (*M* = 42.06, SD = 3.92 months). Their mothers were also involved in the study through the filling in of a short questionnaire. The mothers’ level of education in the sample was: college—4.9%; higher professional education (bachelor, master, specialist)—87%; scientific degree—4.8%. 2.4% of respondents refused to provide information on their level of education.

### 3.3. Measures

#### 3.3.1. Executive functions skills measures

Dimensional Change Card Sort (DCCS; Zelazo, 2006) was performed to evaluate cognitive flexibility. DCCS requires that the child sorts cards; there are three rounds, and rules change for them. First, the classification must be performed based on the color of the picture (al), then the shape (switch trial), and the last round combines contradictory rules: the classification should be based on the color of the shape, depending on the presence of a frame in the picture (post–switch trial). For further analysis, we used the total score (the range consisted of 0–24 points).

Memory for Designs subtest of NEPSY-II (Korkman et al., 2007) was used to evaluate visual–spatial working memory including two parameters: memorization of “images” (the task was to select some pictures following an example from a batch of similar pictures) and memorization of spatial locations of the pictures (children had to remember the exact position of the cards).

Inhibition (Korkman et al., 2007) was used to assesses the ability to inhibit automatic cognitive responses. It includes two series of shapes (circles/squares and arrows). Firstly, the child is asked to name the shape or direction (Naming trial). In the second part of the task, the child is asked to name the shape or direction conversely: to say “circle” when a square is presented and “square” when it is a circle (Inhibition trial). Three scores were taken into analysis: number of corrected errors, number of uncorrected errors, and inhibition processing speed.

#### 3.3.2. Family background

The parent questionnaire was designed with two units. First, it included questions about the socio-demographic characteristics of families (SES and parental education level). Second, it contained questions to obtain data on parents’ toy choice practices in the families of the participating children: (i) What do you focus on when choosing toys to buy for your child? (ii) How do you evaluate the appropriateness of the toy for the age of the child? These questions were asked for an exploratory analysis of the potential relationship between children’s toy selection and their trends-dependent toys buying practices.

### 3.4. Data analysis

The analysis was carried out in three stages. The first step was to describe the most and least popular toys in the both experimental trials. It was performed with using the percentage of the number of choices. It was done using the percentage of the number of toys chosen. In order to illustrate the empirical material, this block of analysis presents children’s answers to the question of why they chose a particular toy. The second step was to determine the proportion of children who chose a trendy toy over the one they had already chosen in the second experimental trial. The third step included an evaluation of the impact of family factors on children’s trend dependency in choosing toys. At the fourth step, a binomial logistic regression was performed to ascertain the effects of age, non-verbal intelligence, and the main EF skills on the likelihood of children having trend dependency in choosing toys.

## 4. Results

### 4.1. Children’s choice of toys in the experimental trials

In the first experimental trial, out of the 12 toys offered, each toy was chosen at least once. Yet, there were toys which had the highest popularity. The frequencies indicate that children most often chose a hospital playset (34.6%), a supermarket playset (20.5%) and a car (10.2%). A dog figure (2.4%) and family animal (0.8%) were chosen less frequently. After choosing a toy, each child was asked to explain why he or she chose that particular toy. Below are the children’s answers to the reasons for the toys that were chosen most often. Children who chose a hospital playset most often explained their decision as follows: “I would play doctor,” “I like to play hospital,” “I like going to hospitals,” “I like being treated,” “I would like to treat others,” “I want to play ambulance and give injections.” Children who chose a supermarket play set were most likely to provide such comments about their decision: “I will sell toys and some fruit,” “I will buy sweets and groceries,” “I often go to the shop with my mum, want to play in it,” “I like to play in the shop.” Children who chose a car mentioned the following reasons for that: “I play it with my dad, mum and friends,” “I can drive, open doors,” “I would play racing with it,” “You can play with the car as if you were a doctor,” “you can go anywhere.” The small group of children who chose a dog said the following about the causes of their choice: “I would play ball with the dog,” “you could play with it as if you were walking,” “I would play vet, treat a dog and feed it.”

In the second experimental trial, children were asked to choose one toy from two: the toy of choice (the first experimental trial) and the Trendy Toy. 43% of the children changed their choice in favor of the trendy toy. Among those children who preferred the viral toy to the toy of choice the most frequent reasons for the choice were: “Because it is scary,” “Because it is very cute,” “Because it is funny and wants to eat people,” “Because I have one at home,” “Because my brother already has one,” “Because it is evil and you can sleep with it,” “Because my mum will not let me buy one,” “Because it is from a cartoon,” “Because it has sharp teeth and big eyes,” “Because its eyes are funny and its mouth is big,” “Because it is scary and the bravest.”

### 4.2. Family factors and child trend dependency in choosing toys

An ANOVA was used to test differences in certain family background factors between two groups of children: who showed trend dependency when choosing toys and who maintained their choice even after being presented with a trendy toy. An ANOVA showed no significant differences between these groups of children on any of the parameters, namely SES, level of parental education and number of children in the family (*p* > 0.05).

The buying of new toys in participating families was mostly based on the wish of the child (39.5%), on the feedback and recommendations of other parents (27.6%). Parents were less often dependent on what they themselves would play with (14.8%) and on the search for information on the developmental benefits of a toy (17.1%). A trendy toy was most frequently selected (21.1%) by children whose parents fully rely on the wishes of the child when buying new toys. However, this relationship was not significant in the correlation analysis (*p* > 0.05).

The assessment of the appropriateness of a toy for a child’s age in the participating families is most often based on common sense and personal experience (69.6%), on package labels (11.4%), on independent search for information about psychological and pedagogical expertise of toys (6.3%). In other cases, parents do not pay attention to the age appropriateness of the toy (7.6%) or are guided by whether their peers have similar toys (5.1%). Correlation analysis found no significant relationship between the way toys are age-appropriate and the child’s exposure to the trend in toy choice (p > 0.05).

### 4.3. EF skills and trend dependency in choosing toys

A binomial logistic regression was performed to ascertain the effects of age, non-verbal intelligence and the main EF skills on the likelihood of children having trend dependency in choosing toys. This model shows what the probability is that the child will reject the chosen toy in favor of the Trendy Toy. Accordingly, the dichotomous dependent variable was whether or not the trend influenced the child’s choice.

The logistic regression model was statistically significant, *χ*^2^(8) = 18.3, *p* = 0.019. The explained variation in the dependent variable based on our model ranges from 23.6 to 31.9%, depending on the Cox and Snell *R*^2^ or Nagelkerke *R*^2^ methods, respectively. Boys were 1.66 times more significantly likely to have trend dependency in choosing toys than girls (see Table 1). Increasing age was not significantly associated with a decreased likelihood that child will reject the chosen toy in favor of the trendy toy. Children with weak inhibitory control were more likely to show trend dependence when choosing toys. Specifically, more uncorrected errors and processing speed in inhibitory control test were significantly associated with an increasing in the likelihood of choosing a Trendy Toy.

**Table 1:** Model coefficients from a binomial logistic regression of the effects of age, non-verbal intelligence, and the main EF skills on the likelihood of children having trend dependency in choosing toys.

		95% Confidence interval				
Predictor	Estimate	Lower	Upper	SE	*Z*	*p*
Intercept	6.6559	−4.0571	17.36889	5.46591	1.218	0.223
Gender:						
girls – boys	1.6652	0.4479	2.88244	0.62107	2.681	0.007
Age in months	−0.1275	−0.3277	0.07267	0.10213	−1.248	0.212
Non-verbal intelligence	−0.0617	−0.2690	0.14553	0.10575	−0.584	0.559
Cognitive Flexibility	−0.1750	−0.5196	0.16956	0.17581	−0.995	0.320
Visual working memory	0.0247	−0.0361	0.08538	0.03098	0.796	0.426
Inhibition corrected errors	−0.1022	−0.4351	0.23080	0.16988	−0.601	0.548
Inhibition uncorrected errors	−0.0878	−0.1716	−0.00402	0.04276	−2.054	0.040
Inhibition processing speed	0.0165	3.71e-4	0.03271	0.00825	2.005	0.045

Estimates represent the log odds of “Toy of choice = Classic toy” vs. “Toy of choice = Trendy Toy”.

## 5. Discussion

The toy industry has been changing rapidly in the last decade due to connection with digitalization and social networks (Plowman and Luckin, 2004; Marsh, 2017). Animated films, video games and viral videos on social media have a huge impact on children’s interests, starting from pre-school age (Adachi and Willoughby, 2017; Edwards et al., 2018). Digital trends quickly become embodied in the physical objects with which children interact in reality. Thus, at certain periods of time, some toys that have gained popularity in media content become “virally” popular among children. The uncontrollability and rapidity of changes in the physical objects with which children play raise a number of concerns. On the one hand, trendy toys may not be age appropriate and relevant to the developmental needs of children. For instance, the Trendy Toy, the subject of this study, provokes fears and psycho-emotional problems in children. This toy has an aggressive appearance and is associated with the horror plots from the video game. On the other hand, trendy toys probably reduce the developmental potential of play because of a decrease in children’s initiative, autonomy in creating play stories.

The findings are divided into three groups, in line with the aims of the study. The first objective of the study was to examine in two-part experiment how children choose toys and how they explain their choices. At first each child had to choose 1 toy out of 12 that he or she would like to play with the most. Each toy in this trial was chosen by the children at least once. The toys with the highest popularity refer to children’s experiences of visiting public places: hospital and supermarket playset. The way the children explained why they chose them is interesting. Almost all answers contain well-defined ideas of how the child would like to play with the toy (see Subsection 4.1). The children had a good understanding of what kind of play actions and plots they would like to perform with the toy. The stories and ideas told by children can mostly be classified as modelling the reality around them in a playful context. But the choice of Trendy Toy in the second experimental trial was motivated by children in quite a different way. Most children described the appearance of the toy (sharp teeth, big eyes), or expressed a desire to have it (for instance “I want it because my brother already has it”). In other words, children’s answers did not contain ideas about what exactly they would like to do with this toy. This pattern of children’s answers when choosing Huggy Wagy supports the concern that play with trendy toys is probably does not provide the full developmental potential. The motivation to choose the Trendy Toy is not related to specific ideas or imaginary situations that the child would like to bring into play.

The second aim was to explore the potential impact of certain family factors on children’s trend dependency in choosing toys. The study did not provide evidence that trend-dependent children differed significantly in SES, parental education level, and number of children from children who were trend-independent when choosing toys. No significant association was also found between the dependency of a child’s trend and the parent’s attitude toward choosing the appropriate age and buying new toys for a child.

The third aim was to assess the impact of EF skills on trend dependency in choosing toys. The results showed that with increasing inhibitory control, children became less likely to depend on trends in their choice of toys. Similarly, as the number of uncorrected errors and processing speed increased the inhibitory control test, children were significantly more often choosing the Trendy Toy. It was also found that boys were 1.66 times more likely than girls to choose a trendy toy. The age of the children was not found to be a significant predictor of trend dependence.

The results support the theoretical concerns stated in the Introduction about the reduced benefits of playing with trend toys for children’s development (Smirnova, 2015; Ryabkova et al., 2019; Veraksa et al., 2020). The findings, on the one hand, point to “empty” motivation in choosing a trend toy: children do not have an understanding of what they will do with the toy. That is, a trend toy, which is not connected with children’s real experience, has no potential to be included in the child’s interesting play stories (Veresov, 2006; Fleer et al., 2017). On the other hand, a trend toy in the present study was more often chosen by children who have difficulties in controlling impulsive cognitive reactions (inhibitory control). According to earlier research, a complete play with a rich story could be useful to support inhibitory control (Veraksa et al., 2019, 2020a,b).

## 6. Limitations and future directions

This study has several limitations. First of all, although trends in toys have become global and are spread throughout the world, attitudes toward them vary depending on the sociocultural values of the region. This study was conducted in Russia, and its results should be interpreted with caution in different sociocultural contexts. Another limitation is the lack of data on how familiar children were with the proposed trendy toy and from which channels information about it came to them. Also, important to note the small amount of information about family factors that could affect the results. Including a detailed survey of parents about their shared values and practices in selecting toys for their child would greatly enhance the results in terms of accuracy and reliability. This study represents the first attempt to examine the problem of children’s dependence on trends. These and other limitations of the study will be addressed in the future.

## 7. Conclusion

The present findings describe the characteristics of children’s toy choices in the context of the spread of global trends. The study was conducted between October and December 2022. During this period, the Huggy Wuggy was a very popular toy in many countries. It is a character from a horror video game, which is a blue monster with sharp teeth. Its goal, according to the plot, is to pursue the player and kill him. Unlike previous virally trendy toys (for instance Pop It or Spinner) this toy has caused a public outcry. There are several reports in the media that the toy has a negative impact on the emotional state of children.

The study is the first to examine children’s dependence on trends in toy choice. The experimental approach with a combination of individual diagnostics allowed one to determine predictors of child’s dependence on trends. Due to the speed and uncontrollability of the spread of new trends in children’s subculture, the results of this study may be of practical value for training the resilience of children to trends that might be harmful to children.

## Data availability statement

The raw data supporting the conclusions of this article will be made available by the authors, without undue reservation.

## Ethics statement

The studies involving human participants were reviewed and approved by the Ethics Committee of Faculty of Psychology at Lomonosov Moscow State University (the approval no: 2022/21). Written informed consent to participate in this study was provided by the participants’ legal guardian/next of kin.

## Author contributions

MG, VS, and NV conceived, conceptualized, designed the study, gathered and analyzed the data, and acquired resources. MG drafted the manuscript. All authors contributed to the article and approved the submitted version.

## Funding

This research was funded by Russian Science Foundation grant number 22–78-10097.

## Conflict of interest

The authors declare that the research was conducted in the absence of any commercial or financial relationships that could be construed as a potential conflict of interest.

## Publisher’s note

All claims expressed in this article are solely those of the authors and do not necessarily represent those of their affiliated organizations, or those of the publisher, the editors and the reviewers. Any product that may be evaluated in this article, or claim that may be made by its manufacturer, is not guaranteed or endorsed by the publisher.
